# γ-H2AX Kinetic Profile in Mouse Lymphocytes Exposed to the Internal Emitters Cesium-137 and Strontium-90

**DOI:** 10.1371/journal.pone.0143815

**Published:** 2015-11-30

**Authors:** Helen C. Turner, Igor Shuryak, Waylon Weber, Melanie Doyle-Eisele, Dunstana Melo, Raymond Guilmette, Sally A. Amundson, David J. Brenner

**Affiliations:** 1 Center for Radiological Research, Columbia University Medical Center, New York, New York, United States of America; 2 Lovelace Respiratory Research Institute, Albuquerque, New Mexico, United States of America; Dana-Farber/Harvard Cancer Institute, UNITED STATES

## Abstract

In the event of a dirty bomb scenario or an industrial nuclear accident, a significant dose of volatile radionuclides such as ^137^Cs and ^90^Sr may be dispersed into the atmosphere as a component of fallout and inhaled or ingested by hundreds and thousands of people. To study the effects of prolonged exposure to ingested radionuclides, we have performed long-term (30 day) internal-emitter mouse irradiations using soluble-injected ^137^CsCl and ^90^SrCl_2_ radioisotopes. The effect of ionizing radiation on the induction and repair of DNA double strand breaks (DSBs) in peripheral mouse lymphocytes *in vivo* was determined using the γ-H2AX biodosimetry marker. Using a serial sacrifice experimental design, whole-body radiation absorbed doses for ^137^Cs (0 to 10 Gy) and ^90^Sr (0 to 49 Gy) were delivered over 30 days following exposure to each radionuclide. The committed absorbed doses of the two internal emitters as a function of time post exposure were calculated based on their retention parameters and their derived dose coefficients for each specific sacrifice time. In order to measure the kinetic profile for **γ**-H2AX, peripheral blood samples were drawn at 5 specific timed dose points over the 30-day study period and the total γ-H2AX nuclear fluorescence per lymphocyte was determined using image analysis software. A key finding was that a significant γ-H2AX signal was observed *in vivo* several weeks after a single radionuclide exposure. A mechanistically-motivated model was used to analyze the temporal kinetics of γ-H2AX fluorescence. Exposure to either radionuclide showed two peaks of γ-H2AX: one within the first week, which may represent the death of mature, differentiated lymphocytes, and the second at approximately three weeks, which may represent the production of new lymphocytes from damaged progenitor cells. The complexity of the observed responses to internal irradiation is likely caused by the interplay between continual production and repair of DNA damage, cell cycle effects and apoptosis.

## Introduction

In the event of an accidental or terrorist incident, the release of radionuclides to the environment is a major concern for acute and chronic exposures. In general, radiation doses resulting from external exposures to ionizing irradiation are more readily assessed than those from radioisotopes incorporated into the body through inhalation or ingestion. Increased information on radiation doses and risk to human health comes from studies on populations exposed to external radiation, while quantitative estimates of the radiotoxicity of internal emitters in humans is limited to a few radionuclides [1]. For internal emitters, the potential heterogeneity of energy deposition in tissues and temporal differences from prolonged exposure times contrasts the relatively uniform and very brief exposures from most external radiation sources/exposures.

Radioactive isotopes of Cesium-137 (^137^Cs) and Strontium-90 (^90^Sr) are considered to be some of the most dangerous radionuclides released into the environment in terms of their high radioactivity, long-lived effects (physical half-lives of about 30 years) and the ease in which they are taken up into the food chain [1–3]. Produced by nuclear fission, both isotopes are of concern in fallout from nuclear weapons and nuclear reactor accidents. Atmospheric atomic weapons testing (predominantly 1950s and 1960s) and the Chernobyl nuclear accident led to wide spread environmental contamination of these nuclear fission by-products in soil, water and vegetation [4,5]. Radionuclide waste contamination of the Techa River by the Mayak nuclear weapons facility in the South Urals exposed thousands of people living in rural villages along the river to protracted internal and external exposures to ionizing radiation [6]. Recent epidemiological studies have estimated the dose-response relationship for leukemia risk [7] and solid cancer mortality [8] in the Techa River cohort. Due to widespread medical and industrial usage, ^137^Cs and ^90^Sr isotopes pose a high risk for incorporation into an improvised nuclear device (IND) or a radiological dispersal device (RDD) or “dirty bomb” [9]. The ^137^Cs-based radiological accident at the city of Goiânia in central Brazil illustrates the catastrophic effects of large scale environmental radioactive contamination from a loss of control of a radiotherapy source (housing about 100 g CsCl_2_) stolen from an abandoned hospital site radioactive contamination [10].

The biochemical and physical properties of the ^137^Cs and ^90^Sr radionuclides contribute to their unique temporal pattern and biological behavior. Cesium and its salts are highly soluble in water. With similar chemical and physical properties as potassium, ^137^CsCl is rapidly absorbed from the gastrointestinal tract or lungs and permeates the entire body providing relatively uniform protracted beta particles and gamma irradiation [2,11]. Biokinetic models in adults show that ^137^Cs is eliminated fairly quickly from the body through the urine, such that approximately 10–15% of the isotope intake is excreted within 2–3 days with the remainder cleared by ~90 days [12,13]. In contrast, ^90^Sr chemically resembles calcium and is readily incorporated into bones and teeth, irradiating the bone marrow and soft tissues surrounding the bone. As ^90^Sr decays, it releases moderate energy beta particles (maximum of 0.5 MeV) forming yttrium-90 (Y-90), which in turn emits strong, energetic beta-particles (maximum of 2.3 MeV) and forms stable zirconium. The biological half-life of ^90^Sr is longer than that of ^137^Cs because only about 70–80% of it passes through the body with the remaining deposited and trapped in bone (20–30%) or distributed among the blood volume, extracellular fluid, soft tissue, and bone surface (1%), where it may stay and decay or be excreted (Environmental Protection Agency; http://www.epa.gov/radiation/radionuclides/strontium.html). Since ^90^Sr can be stored in bone for many years, there is the increased risk of carcinomas of the bone and leukemia [14,15]. It has also been proposed that Strontium-90 can chemically bind to chromosomes [16].

In the present study we have developed two mouse models for: a) chronic relatively uniform whole-body irradiation using intraperitoneal, systemically distributed ^137^CsCl as a radiation source, and b) chronic non-uniform low-LET radiation resulting from parenterally administered liquid soluble ^90^SrCl_2_. Based on known biokinetics for ^137^CsCl and ^90^SrCl_2_ [17–20], a single injection activity was calculated to produce accumulated total-body absorbed doses of up to 10 Gy over 30 days of protracted ^137^CsCl exposure and for ^90^SrCl_2_, skeletal absorbed doses were calculated over two dose ranges, a lower dose range from 0 to 5 Gy and a much higher dose range up to approximately 49 Gy over a similar 30 day period. To evaluate the ionizing radiation-induced DNA damage effects in peripheral mouse blood lymphocytes, we used the established biodosimetry biomarker γ-H2AX [21–23] and indirect immunofluorescence protocols to measure the induction and repair of nuclear DNA double strand breaks (DSBs). In response to ionizing radiation exposure, the histone H2AX is rapidly phosphorylated at serine 139 and phosphorylated H2AX (γ-H2AX) molecules form foci at or near the vicinity of DNA DSBs. The rapid formation of the γ-H2AX protein has been shown to be sensitive and linear with absorbed dose over a wide dose range [22,24,25]. To assess the DNA DSB repair capacity in peripheral blood C57BL/6 mouse lymphocytes, total nuclear γ-H2AX fluorescent yields were measured at specific time points over a 30 day study period following a single administered activity of ^137^CsCl and ^90^SrCl_2_. The doses of the two internal emitters as a function of time post exposure were verified by real-time dosimetry. Presented are the *in vivo* γ-H2AX kinetics patterns as a function of elapsed time post injection of each radionuclide. A mechanistically-motivated model was used to analyze these data to quantify and interpret the temporal pattern of γ-H2AX fluorescence.

## Methods and Materials

### Animals

The animal studies were conducted in accordance with applicable federal and state guidelines and were approved by the Institutional Animal Care and Use Committee (IACUC) of the Lovelace Biomedical and Environmental Research Institute (LBERI) and the IACUC Columbia University (approval number AC-AAG4356). Male C57BL/6 mice (approximately 10–12 weeks old, 25–30 g) were purchased from Charles River Laboratories (Frederick, MD) and quarantined for 14 days prior to group assignment by body weight stratification for randomization onto the study.

The animals were divided into five radiation dose groups, 8 animals per group. For the Cesium-137 study, mice were injected intraperitoneally (IP) with a single 8.0 ± 0.3 MBq activity ^137^CsCl solution in a volume of 50 μl. For the Strontium-90 study, animals were administered intravenously (IV) by tail vein injection with 1.55 MBq ± 0.1 (high dose) and 200 ± 0.3 kBq (low dose) ^85/90^SrCl_2_ solution in a volume of 50 μL. Strontium-85 (^85^Sr) was used as a photon-emitting tracer for the purpose of measurement of strontium whole-body content. Strontium-85 comprised approximately of 1% of the total strontium activity. Although the addition of Sr-85 contributed gamma rays to the emitted radiation from Sr-90/Y-90, the photons added less than 0.01% to the absorbed radiation dose to bone and bone marrow. Each mouse was individually weighed just prior to group assignment and radioisotope injection. After intravenous administration of the radionuclides, the ^137^Cs and ^85^Sr/^90^Sr injected mice were housed individually in microisolator cages with lead shielding to avoid radiation exposure due to cross-irradiation from adjacent mice. The control animals were left untreated and housed four mice per cage. All animals had unlimited access to Teklad Certified Global Rodent Diet 2016 (Harlan Teklad, Madison, WI) and water except during radionuclide administration and whole-body *in vivo* counting. During the study, the mice were observed twice daily for any signs of acute toxicity characterized by hunched posture, lethargy, and inactivity. There were no unusual or adverse effects related to the study noted for any intravenously-injected animals, although the high-dose ^90^Sr mice exhibited some localized irritation at the injection site.

### Biokinetics and Dosimetry

Whole body ^137^Cs and ^85^Sr/^90^Sr content was measured using the LBERI *in vivo* photon counting system described previously in more detail in our companion studies [26–29]. Briefly, LBERI utilizes a pair of dual-scintillator [NaI(Tl)-CsI(Tl)] crystal detectors. The system is calibrated by creation of size specific phantoms which are used to generate linear curves to which the study samples are compared in order to give the amount of radioactivity present in each sample. The ^137^Cs and ^90^Sr whole-body retention profile *in vivo* measurements were obtained by serial counting for each mouse and normalized to the amount of ^137^Cs and ^90^Sr present in each animal on day 0. The amount of radioactivity present in each animal was measured daily on days 0–7 and then on days 10, 14, 17, 20, 23, 27 and 30 after ^137^Cs administration and on days 9, 12, 16, 20, 25, 27, and 30 (high-dose ^90^Sr) and 9, 11, 15, 16, 20, 23, 25, 27, and 30 (low-dose ^90^Sr) after ^85^Sr/^90^Sr administration. Each day prior to sample analysis, the counting system was calibrated using ^137^Cs and ^85/90^Sr NIST-traceable standard solutions.

The calculation of radiation committed absorbed doses for both radionuclides were based on the average whole body retention equation of each animal group. In order to convert the empirical ^137^Cs and ^90^Sr retention profile data into a committed absorbed dose, dose coefficients were derived for each specific sacrifice time. The calculation was based on specific absorbed fractions for electrons and photons (S values) published by Stabin et al. [30]. These S values were developed specifically for young adult mice and rats, to convert the deposition of photon and electron energy from radioactive decay and radioactive progeny into a dose coefficient (dose in Gy per unit of administered activity), and integrated over the experimental lifespan from radionuclide injection to sacrifice. The committed absorbed doses were calculated by multiplying the dose coefficient (Gy Bq^-1^) related to the specific sacrifice time for each animal in the study by the administered activity (Bq). The ^137^CsCl and ^90^SrCl_2_ formulations are highly soluble. Since the biological distribution of ^137^Cs is relatively uniform throughout the body, only the whole-body committed absorbed dose was calculated and used for this study. Since ^90^Sr is a bone-seeker and is deposited mainly in bone, most of the committed dose is delivered to skeleton tissue and bone marrow.

### Sample Collection

For each radionuclide study, the same amount of radioisotope activity was injected into each animal, and the euthanasia time points post-injection were selected to result in the delivery of specific absorbed doses to the whole body/skeleton of the mice. On scheduled necropsy days, the mice were euthanatized by intraperitoneal (IP) injection of Euthasol (> 150 mg/kg [390 mg/mL pentobarbital and 50 mg/mL phenytoin in sterile saline]) and weighed. Whole blood was collected by cardiac puncture into microtainer tubes with lithium heparin (#365971; BD Becton Dickinson & Co, Franklin Lakes, NJ). Blood samples were collected at five specific data points: on Day 2, 3, 5, 20, and 30 after administration of ^137^CsCl and Day 4, 7, 9, 23 and 30 and 4, 7, 9, 25 and 30 after high and low dose ^85^Sr/^90^Sr administration, respectively. For each time/dose point 8 irradiated mice and 8 control mice were sacrificed. Since we assume a homogenous distribution for ^137^CsCl in the tissues, only whole body measurements were performed, whereas for ^90^SrCl_2_, a full necropsy was conducted with ^90^Sr content determined in the liver, spleen, kidneys, lungs and trachea, muscles (right and left quadriceps), gastrointestinal tract (stomach and esophagus, upper and lower intestine), testes, femurs, and all soft tissue remains were collected. The brain and eyes were removed from the skull and combined with the soft tissue remains. The carcass was analyzed separately.

### Lymphocyte Isolation and Measurement of γ-H2AX yields

Peripheral blood mouse lymphocytes were isolated by ficol gradient. Whole blood samples, (~ 100–200 μl) were diluted with 5 volumes of RPMI-1640 medium (Invitrogen, Eugene, OR) and carefully layered over an equal volume of lymphocyte separation media (Histopaque-1083; Invitrogen). The samples were centrifuged at 1300 RPM for 30 min to form a lymphocyte band at the interface between the separation medium and plasma. The freshly isolated lymphocytes were washed twice with phosphate buffered saline (PBS) and fixed with ice-cold methanol for a minimum of 30 minutes and stored in suspension at 4°C. To prepare the slides, the cells were spun and all the fixative supernatant was removed leaving ~ 100 μl in order to resuspend the cells. The total cell suspension was added drop wise onto a microscope slide and allowed to air-dry. For the immunodetection of γ-H2AX, the cells were blocked with 3% bovine serum albumin (BSA; Sigma, St Louis MO) for 30 minutes at room temperature and incubated with a rabbit polyclonal γ-H2AX (phospho S139) antibody (dilution 1:600; #ab2893 Abcam Inc., Cambridge, MA) for 2 h at room temperature. Following 3 washes with PBS, the cells were visualized with a goat anti-rabbit Alexa Fluor 488 (AF488) secondary antibody (dilution 1:1000; Invitrogen) and washed 3 times with PBS and the nuclei were counterstained with DAPI (Vectashield® mounting medium with DAPI (#H-1200; Vector Laboratories, Burlingame, CA). Images of cells were obtained using an Olympus epifluorescence microscope (Olympus BH2-RFCA). Fluorescent images of DAPI-labeled nuclei and AF488-labeled γ-H2AX were captured separately for each absorbed dose using an Olympus epifluorescence microscope (Olympus BH2-RFCA) and 60x oil immersion objective. Quantification of γ-H2AX yields was determined by measuring the total γ-H2AX nuclear fluorescence per lymphocyte and analyzed using image analysis software [25]. Advanced apoptotic cells observed as a gross change in morphology of the DAPI-labeled nuclei were not included for analysis by the software program. The fluorescence data, generated in Excel format as the average pixel value for every scored nucleus was edited for high intensity **γ**-H2AX fluorescence labeling (average fluorescence >2/3 maximum fluorescence) due to fragmented nuclear DNA [31]. For data acquisition, ~ 200 paired lymphocyte images were captured and analyzed per data point.

### Statistics

The probability distribution of cellular γ-H2AX fluorescence values was well approximated by the stretched exponential (Kohlrausch) function [32]. The mathematical form of the cumulative probability distribution was: P_cum_ = 1 –exp[-*a* f ^*b*^], where f is **γ**-H2AX fluorescence, and *a* and *b* are adjustable parameters. Confidence intervals for parameters *a* and *b* were produced by fitting the expression for P_cum_ to 10,000 random data sets produced from the original data set by nonparametric bootstrapping. Based on the shape of the distribution of cellular **γ**-H2AX fluorescence values, we selected the 50^th^, 75^th^ and 90^th^ percentiles as input data to assess the temporal kinetics of γ-H2AX fluorescence after administration of radioactive material. The standard deviation of each of these percentiles was estimated by nonparametric bootstrapping of the γ-H2AX fluorescence data, using 10,000 replicates. Background **γ**-H2AX fluorescence measured in control samples was subtracted from the signal measured in irradiated samples, producing the excess signal. Because the intensity of the fluorescence was measured in arbitrary units which precluded direct comparison between different radiation absorbed doses, for the evaluation of temporal kinetics we normalized the maximum of the excess γ-H2AX signal to unity. This allowed data from all selected percentiles to be pooled and fitted by a common formalism.

The normalized γ-H2AX fluorescence data (Nf) were reasonably described by the “two-peak” function Nf = exp[-k_1_ (t–t_1_)^2^] + exp[-k_2_ (t–t_2_)^2^], where t is time after administration of radioactive material, and k_1_, k_2_, t_1_ and t_2_ are adjustable parameters. The mechanistic interpretation for this formalism is as follows: We hypothesize the γ-H2AX signal is first observed in mature lymphocytes. The peak of their γ-H2AX response occurs around time t_1_. As time increases beyond t_1_, DNA repair processes and/or radiation-induced death of the mature lymphocytes (represented by parameter k_1_) reduce the γ-H2AX signal. However, at even later times (around t_2_), a new peak of γ-H2AX fluorescence is produced by new lymphocytes, which were produced from radiation-damaged stem and/or progenitor cells. The signal intensity in this second peak is also affected by processes of DNA repair and/or cell death, represented by parameter k_2_.

## Results

**Fig 1:**
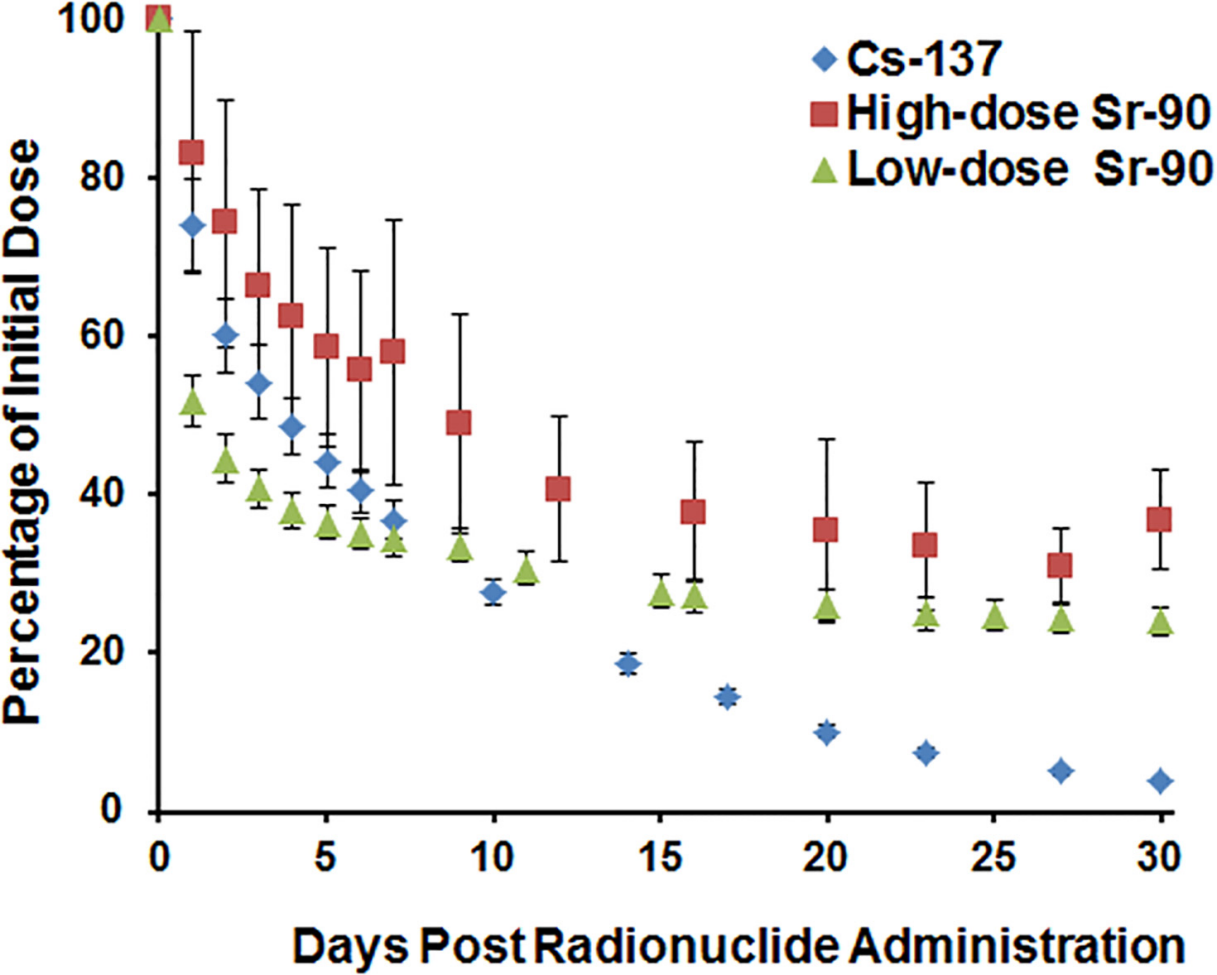
Retention profiles for ^137^CsCl and ^90^SrCl_2_.

Biokinetics and dosimetry measurements for both the radionuclides were calculated based on the whole-body retention data. Fig 1 shows the whole-body counting data normalized to the amount of ^137^Cs and ^90^Sr present in each animal on Day 0 following injection. Table 1 shows the accumulated total body absorbed doses (Gy) calculated at specific time points following isotope injection. The biokinetic and dosimetric modeling showed that within the first week, more than 60% of the initial (8.0 ± 0.3 MBq) ^137^Cs activity and ^90^Sr (low dose; 200 ± 0.3 kBq) have been excreted, whereas ~ 60% remained after injection with (high dose; 1.55 ± 0.1 MBq) ^90^Sr. By Day 30, 96% of ^137^Cs was excreted whereas 37% and 24% remained in the skeleton for ^90^Sr (high dose) and ^90^Sr (low dose), respectively. Table 1 also highlights the changing dose rates for the accumulation of the radionuclides over the 30-day study time. The results show that the dose rate is more or less proportional to the administered isotope activity. For ^137^Cs, the dose rate decreased with increasing elimination from the body. The approximate average dose rate of accumulation between Day 3 and Day 5 was 0.52 mGy/min whereas between Day 5 and 20 the average dose rate had dropped by ~50% to 0.25 mGy/min leading to a further 90% drop by Day 30. For high-dose ^90^Sr, the accumulation rate increased up to Day 9, after which time there was an approximate 60% drop in the rate by Day 23 with no further accumulation by Day 30. In contrast, for low-dose ^90^Sr, there was no apparent decrease in the dose rate between Day 7 and Day 25.

**Table 1:** Total Body Absorbed Doses (Gy ± SD) and dose rate (mGy/min) of ^137^Cs and ^90^Sr over the 30-day period.

DAY	Cs-137	Rate	DAY	Sr-90 (High)	Rate	DAY	Sr-90 (low)	Rate
	(Gy ± SD)	(mGy/min)		(Gy ± SD)	(mGy/min)		(Gy ± SD)	(mGy/min)
**2**	2.0 ± 0.1	0.67	**4**	11.4 ± 2.0	1.98	**4**	1.2 ± 0.1	0.21
**3**	2.7 ± 0.4	0.52	**7**	20 ± 1.9	2	**7**	1.8 ± 0.1	0.14
**5**	4.1 ± 0.4	0.5	**9**	28.5 ± 4.1	2.92	**9**	2.1 ± 0.3	0.11
**20**	9.5 ± 0.4	0.25	**23**	49.4 ± 11.2	1.04	**25**	4.8 ± 0.4	0.11
**30**	9.9 ± 1.2	0.03	**30**	49.4 ± 12.0	0	**30**	5.3 ± 0.7	0.07

Overall, measurements of low-dose ^90^Sr activity in the tissue and whole-body samples showed the skeleton retained about 95% of the total whole-body activity for all time periods. At the earlier sampling times, small amounts of activity were generally observed in most tissues but as the study progressed, the amounts present decreased to undetectable levels. All animals displayed soft tissue content at the 4-day necropsy with an average burden of 0.36% of the recovered activity (RA), which decreased at the 7-day necropsy to 0.31% RA and 0.24% RA at the 30-day time point. Carcass content was stable through the duration of the study ranging from 91.4% RA (day 4) to 93.7% RA (day 25). This is in contrast to the high-dose ^90^Sr mice where the carcass content steadily increased to 79% RA at day 9 before decreasing for the remainder of the study to a minimum of 30.6% RA by the conclusion of the study. High levels of ^90^Sr activity was also measured in the femur which ranged from 2.7% RA (day 7) to 1.6% RA (day 30) compared to 6.7% RA (day 4) and 5.7% RA (day 25) measured in the low-dose ^90^Sr animals.

### γ-H2AX Kinetic Profile

**Fig 2:**
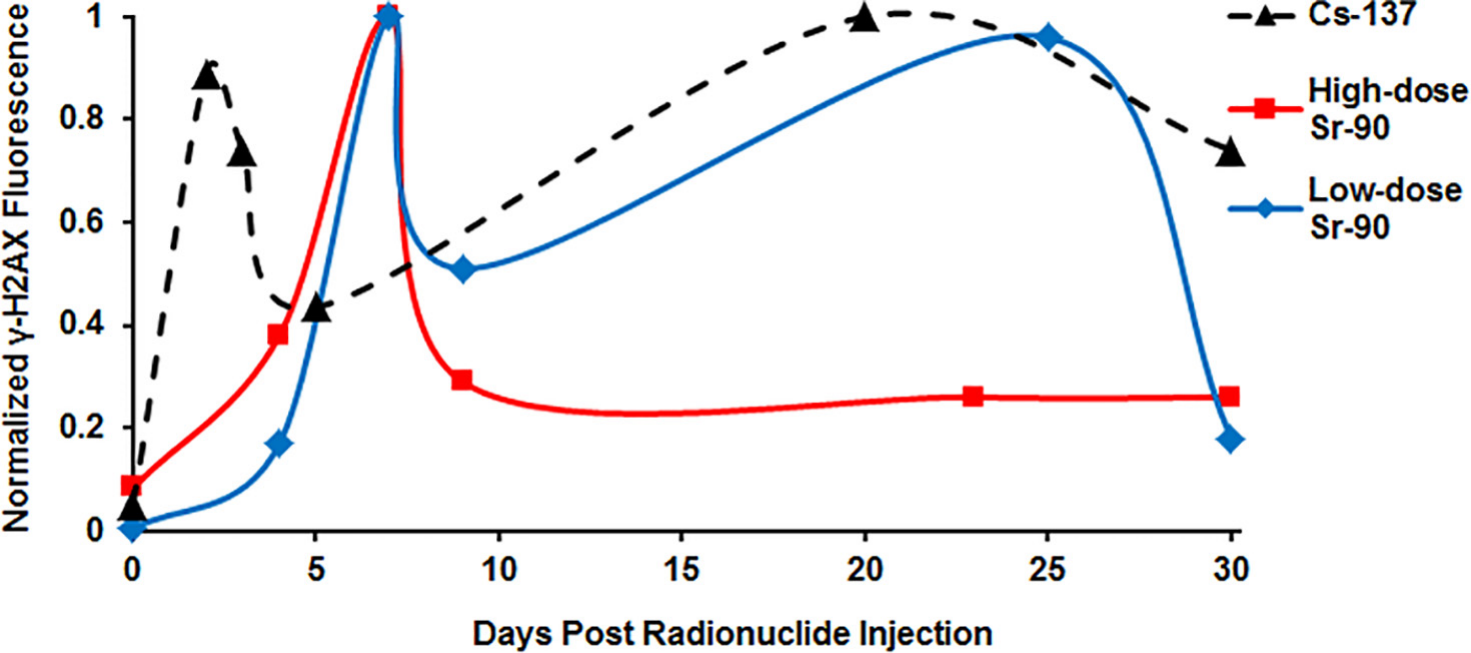
Median response pattern of γ-H2AX yields in peripheral blood mouse lymphocytes following 30-day internal exposures to ^137^Cs (dashed black line), high-dose ^90^Sr (solid red line) and low-dose ^90^Sr (solid blue line). The curves connecting the points are splines shown for convenience only, to guide the eye.

Gamma H2AX foci were detected by indirect immunostaining and quantified by fluorescence intensity relative to the unirradiated control cells. Fig 2 shows the median γ-H2AX nuclear fluorescence values measured in lymphocytes at the specific time/dose points following the injection of the radionuclides. Because the fluorescence was recorded in arbitrary units, comparison of the γ-H2AX kinetics was assisted by normalizing the median γ-H2AX values for each radionuclide experiment as follows: each data point was divided by the highest value observed in the given experiment. For example, the highest median fluorescence value used for normalization occurred at day 7 for Sr (both high and low doses) and at day 4 for Cs. The γ-H2AX kinetic profile for ^137^Cs exposure (denoted by black dashed line) indicated that the γ-H2AX yields at Day 2 (total body committed absorbed dose = 1.95 Gy) showed the highest levels followed by a rapid decline in γ-H2AX frequency by Day 5 (total body committed absorbed dose = 4.14 Gy), after which time there is a gradual increase in the γ-H2AX frequency up to 30 Days. Although the results show that at Day 5 the γ-H2AX yields are significantly above non-irradiated baseline levels, the fact that there are no blood draws between Day 5 and Day 20 makes it difficult to determine at what the point the γ-H2AX yields continue to decrease or when they start to increase. For the ^90^Sr exposures, the high-dose ^90^Sr study data (denoted by solid red line) show that γ-H2AX yields appear to increase up to Day 7 (total skeletal committed absorbed dose = 20 Gy), after which there is a rapid decease in γ-H2AX levels by Day 9 (total skeletal committed absorbed dose = 28.5 Gy) with no apparent further increase over the remainder of the study. The γ-H2AX kinetic profile for low-dose ^90^Sr exposure (denoted by solid blue line) similarly showed that the total γ-H2AX fluorescence levels continue to increase up to Day 7 (total skeletal committed absorbed dose = 1.8 Gy), after which time there is apparent 50% drop in γ-H2AX levels. At some point between Day 9 and Day 25 the γ-H2AX protein levels started to increase leading to drop off in yields by Day 30. For all three studies, the median γ-H2AX values for the non-irradiated control lymphocyte samples measured over the 30-day study period are plotted at the time zero, 0 Gy data point.

### Mathematical modeling

**Fig 3:**
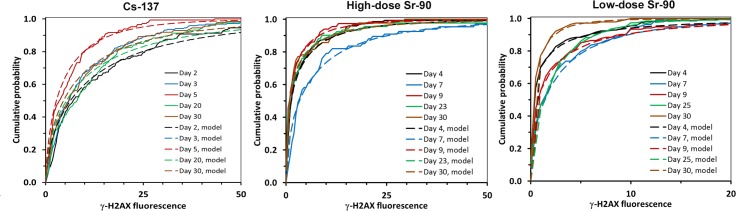
Observed and fitted cumulative probability distributions of cellular γ-H2AX fluorescence values at different times after administration of radioactive material. A shift to the right represents a “peak” of the fluorescence signal and a shift to the left, a trough.

For all analyzed radionuclide doses and times after administration, the cumulative probability distribution of cellular γ-H2AX fluorescence values was well approximated by a stretched exponential function [32] described in Materials and Methods. Both ^137^Cs and ^90^Sr the best-fit values of the shape parameter *b* were always less than unity (Table 2). Consequently, the function reproduced the following observed data patterns: (1) the modal γ-H2AX fluorescence value was equal to zero, i.e. many cells had no fluorescent foci at all even when a large radionuclide dose was accumulated; (2) there was a prominent “tail” of the probability distribution extending towards large fluorescence values (Fig 3), i.e. quite a few cells had large numbers of fluorescent foci per cell. This “tail” may represent cells that are likely to proceed to apoptosis. Examination of these cumulative probability distributions suggested that the temporal kinetics for γ-H2AX fluorescence after administration of either radionuclide were not monotonic, but exhibited two “peaks” separated by a “trough”. For ^137^Cs, the trough occurred on Day 5 after administration–the cumulative probability distribution for that day was visibly shifted to the left, towards smaller fluorescence values (red line in ^137^Cs panel of Fig 3). Consequently, the two peaks occurred before and after the trough. For ^90^Sr the trough was not so apparent, but there was a clear peak on Day 7 (i.e. a shift of the cumulative probability distribution to the right) at both low and high doses (blue lines in ^90^Sr panel of Fig 3).

**Table 2:** Best-fit parameters for the cumulative probability distributions of cellular γ-H2AX fluorescence values at different times after administration of radioactive material. Details are described in the main text.

Radionuclide	Time after admini- stration (days)	Parameter *b*		
		value	95% CIs	
^137^Cs	2	0.62	0.59	0.67
	3	0.62	0.59	0.68
	5	0.67	0.60	0.76
	20	0.61	0.59	0.66
	30	0.62	0.58	0.68
^90^Sr, low dose	4	0.38	0.36	0.41
	7	0.61	0.59	0.66
	9	0.47	0.44	0.52
	25	0.75	0.71	0.80
	30	0.53	0.48	0.59
^90^Sr, high dose	4	0.52	0.50	0.57
	7	0.59	0.55	0.64
	9	0.51	0.41	0.56
	23	0.46	0.42	0.51
	30	0.46	0.43	0.49

As visualized by the probability distributions shown in Fig 3, the γ-H2AX fluorescence was not normally distributed, but instead had a very asymmetric distribution with a large “tail”. This behavior was likely caused by the interplay between continual production and repair of DNA damage, cell cycle effects and apoptosis. Because of the shape of the distribution, we focused on analyzing the median γ-H2AX fluorescence, rather than the mean, because the mean is more sensitive to fluctuations and outliers. For example, when 3 (out of 155) of the highest fluorescently labeled cells are excluded from the low-dose ^90^Sr Day 9 data point, the mean total γ-H2AX fluorescence level drops by 22% whereas the median only by 2%. As a result, modeling of the kinetics data was based on the median γ-H2AX values.

**Fig 4:**
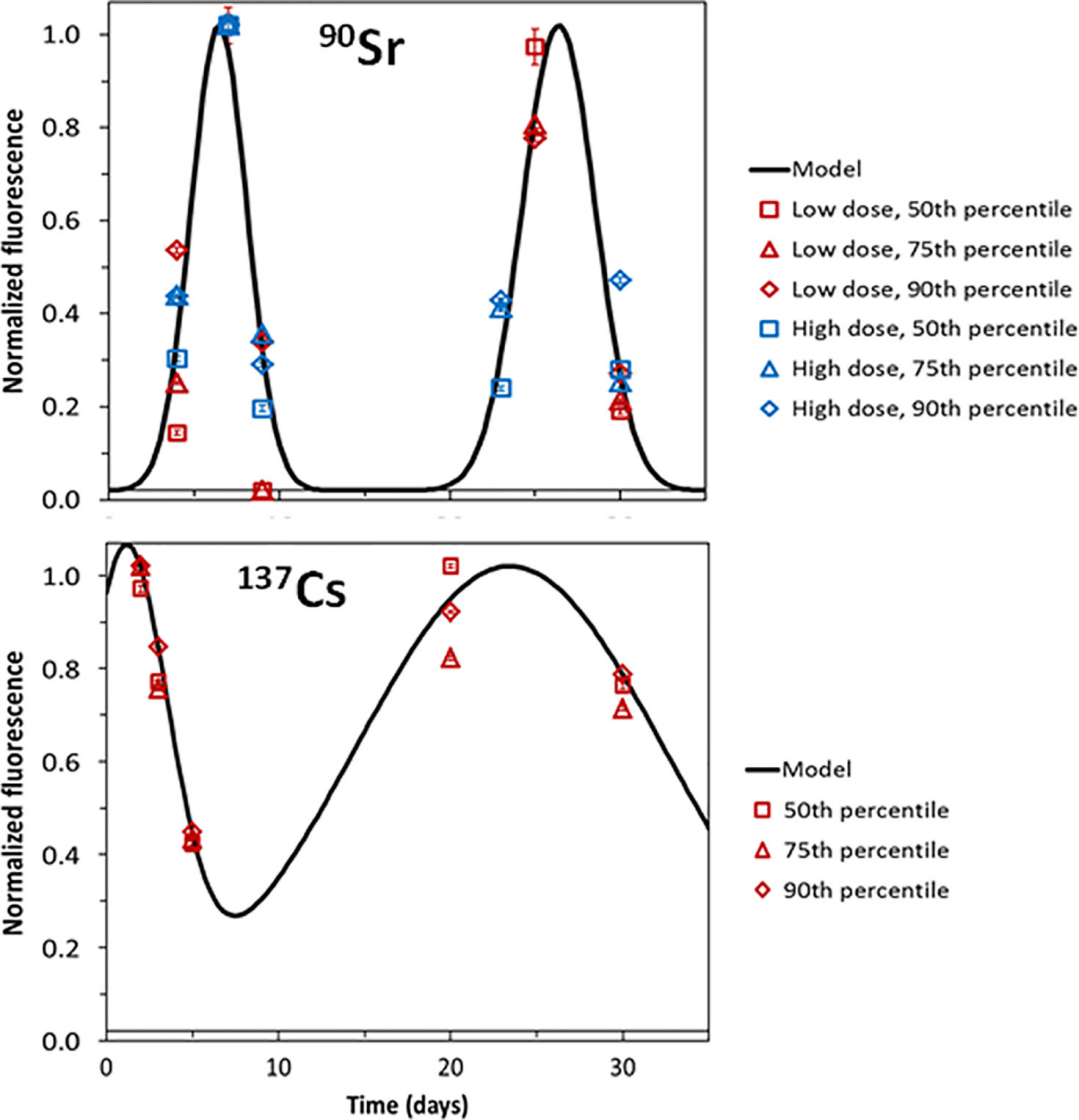
Temporal kinetics of γ-H2AX fluorescence after single administration of high and low dose ^90^Sr, and ^137^Cs. Error bars represent standard deviations.

Table 3 and Fig 4 show a more detailed analysis of γ-H2AX fluorescence kinetics which supports the presence of two peaks at early and late times. The value of the 50^th^ percentile (the median) indicates that 50% of the cells had fluorescence below this value. Correspondingly, the 75^th^ and 90^th^ percentile values indicate that 75% and 90% of cells have fluorescence below these values. For ^137^Cs, the first peak occurred at the shortest observation time, whereas for ^90^Sr it occurred at ~ Day 7 post-injection. The second peak occurred at Days 23–26 for both radionuclides. It is important to note that given the absence of blood draw time points between the two peaks (i.e. between days 9 and 23), the γ-H2AX kinetics within this time period were not observed and remain unknown. Future research is needed to test the two-peak interpretation of the γ-H2AX kinetics during protracted internal emitter exposure, which may display even more complex patterns. The initial decline in γ-H2AX yields and the subsequent trough between peaks may represent the death of mature lymphocytes that were present at the start of radiation exposure, whereas the second peak may represent the contribution of newly formed lymphocytes, which have been produced from radiation-damaged stem and/or progenitor cells.

**Table 3:** Best-fit parameter values for the cumulative probability distribution of cellular γ-H2AX fluorescence values.

Parameter (days)	Interpretation	Best-fit value	95% CIs		Best-fit value	95% CIs	
		^90^Sr			^137^Cs		
t1	peak of signal from old cells	6.46	6.43	6.48	1.09	1.03	1.15
k1	standard deviation of peak around t1	1.64	1.62	1.66	2.52	2.47	2.57
t2	peak of signal from new cells	26.38	26.33	26.43	23.43	23.26	23.61
k2	standard deviation of peak around t2	2.16	2.13	2.20	9.00	8.72	9.30

## Discussion

It is widely accepted that double strand breaks (DSBs) constitute one of the most important types of DNA damage caused by ionizing irradiation and other genotoxic agents because DSBs are more difficult to repair than many other lesions, and incorrect repair of DSBs (e.g. misrejoining of broken DNA strands from different chromosomes) can result in cytotoxic or carcinogenic genomic alterations [33]. The occurrence of radiation-induced DNA DSBs has been extensively studied by biochemical or cell imaging techniques [34]. Virtually all studies performed to date have relied on acute delivery of radiation dose from external radiation sources. As far as we are aware, no previous studies have used DNA repair biomarkers to examine DNA repair capacity following exposure to the protracted ionizing radiation effects of an internal emitter. To this end, we used the γ-H2AX biodosimetry marker to examine the effect of uniform (^137^Cs) and non-uniform (^90^Sr) protracted internal radiation exposure on the *in vivo* induction and repair of DNA DSBs in mouse peripheral blood lymphocytes.

Biokinetic and dosimetric modeling of the radionuclides showed that the internal dose of the radionuclides covered a dose range important for triage (2 to 10 Gy) with very high total body doses achieved in the early sampling times after high dose ^90^SrCl_2_ administration (Table 1). The retention profiles (Fig 1) highlighted the differences between the two isotopes such that by Day 30, ^137^CsCl was almost completely eliminated in the urine whereas 24–37% ^90^SrCl_2_ was retained in the skeleton. Radionuclide activity is reduced through excretion in the urine, the total body dose accumulated at a lower rate. Fig 2 shows the γ-H2AX kinetic profile pattern of γ-H2AX signal as a function of elapsed time in peripheral mouse lymphocytes for the two internal emitters. The rapid increase in lymphocyte γ-H2AX levels 2 days after administration of the ^137^Cs indicated that the isotope quickly distributed within the tissues. Despite the increasing total-body committed absorbed dose (Table 1), the results show that by Day 5 the γ-H2AX frequency had significantly decreased, after which time the γ-H2AX yields began to increase up to Day 20. As the rate of accumulation of the ^137^Cs decreased, the apparent lack of dose response for the formation of γ-H2AX suggests that the rate of γ-H2AX disappearance is not dependent on dose rate. There is a significant γ-H2AX signal 4 weeks after initial exposure compared to the unirradiated control lymphocytes. The ratio of total γ-H2AX fluorescent yields measured in the ^137^Cs-irradiated mouse lymphocytes relative to the unirradiated control cells was larger than for the ^90^Sr-exposed mice. This is not surprising given the fact that as a potassium analogue, ^137^Cs is likely to be more uniformly distributed throughout the body, thus a larger proportion of circulating blood lymphocyte cells will likely be continually exposed to random ionizing radiation gamma rays over the 30-day study period.

Radionuclide tissue content following ^90^Sr exposure indicated that a large proportion of the isotope predominantly localized in bone tissue following injection and over the time course of the 30-day study. The γ-H2AX kinetic profile pattern after ^90^Sr exposure suggested that the peak lymphocyte DNA DSB damage was observed at Day 7, after which the γ-H2AX frequency rapidly decreased with the γ-H2AX levels in lymphocytes exposed to high-dose ^90^Sr reduced by a larger percentage. Although the committed absorbed doses for high-dose ^90^Sr mice were almost 10 fold higher than the low-dose ^90^Sr mice, the total γ-H2AX yields measured within the first week were only 3 fold larger, indicating that the response is not linear or dependent on dose. Overall, the ^90^Sr γ-H2AX study data show persistent DNA damage over the 30-day study period. Here we may speculate that that the electron scatter from the localization of the isotope to the bone surface has exposed a large proportion of the circulating blood cells as they enter the irradiated regions. We might further speculate that given the fact that the ratio of the range of the particle to the diameter of the whole mouse is much larger than for human, the high-energy β particles (maximum of 2.28 MeV) emitted from yttrium-90 decay could have travelled distances up to 1cm [35] and exposed the local circulating blood lymphocytes to a range of radiation doses leading to reduced DNA repair due to a higher density of lesions and multiple damaged sites.

The cumulative probability distributions of γ-H2AX fluorescence (Fig 3) were qualitatively similar for both radionuclides, and at all investigated doses–they were well described by a stretched exponential function with a long “tail” of lymphocytes with high levels of γ-H2AX. These results may be a consequence of the complexity of DSB repair kinetics (multiphasic rather than mono-exponential) and/or of the protracted delivery of radiation dose. The continual bombardment of gamma and beta particles to induce new DNA damage, the constant changing of dose and dose rates, the effects of cell cycle repair and apoptosis all add to the complexity of the radiation-induced cellular effects posed by the inhalation or ingestion of an internal emitter. Analysis of the ^90^Sr and ^137^Cs data using a mathematical model suggested that the temporal kinetics of γ-H2AX foci after radionuclide administration were consistent with two distinct peaks: the first occurred during the first week, and the second occurred about 3 weeks later (Fig 4). Although, a general two wave response pattern holds for both isotopes, the specifics are likely due to their distribution within the mouse and to the energy of the emitted photons or electrons.

For ^90^Sr, where two dose ranges were used and which differed by a factor of 10, there was surprisingly no major difference in the temporal kinetics of γ-H2AX foci, and the combined data from both doses could be described by the same parameters (Fig 4, Table 3). This suggests that even the lowest tested dose of ^90^Sr was sufficient to cause DNA damage and death of mature lymphocytes, and these processes (which constitute the first peak of γ-H2AX fluorescence) took several days to manifest themselves. Increasing the ^90^Sr dose 10-fold did affect the timing of the first γ-H2AX fluorescence peak. Based on our mechanistic model interpretation, we hypothesized that the second (delayed) peak was possibly produced by new lymphocytes generated from irradiated progenitors.

When the high dose ^90^Sr exposure data were examined separately from the low dose data, the second peak was not observable. This effect may be due to chance–for example the low dose Sr data contained a measurement at 25 days, close to the predicted maximum of the second peak, whereas the high dose data did not contain a measurement at the same time. Alternatively, the absence of the second peak may be due to the fact that the very high skeleton doses resulted in increased cell killing and death of the hematopoietic stem cells, thereby affecting the production of newly formed “damaged” cells. It may also be possible that given the timing of the mouse sacrifice and blood draws, the timing for the generation of the newly formed “damaged cells shifted.

The data for ^137^Cs were consistent with the same interpretation of early and delayed γ-H2AX fluorescence peaks. The main differences from ^90^Sr data were an earlier onset of the early peak, and an increased “width” of the delayed peak (Fig 4, Table 3). These differences were perhaps due to the fact that ^137^Cs, and the DNA damage induced by its radiation, were distributed more homogeneously throughout the body and perhaps within the tested dose range, the timing was mostly determined by cell turnover/apoptosis rates, rather than by the amount of DNA damage. This suggests that radiation-induced hematopoietic cell death is likely to play an important role in contributing to the γ-H2AX yields. Previous studies including our own have shown that mouse T-cell lymphocytes are radiosensitive to low doses of X-rays [23,36] and that the decrease in T-cell counts post-irradiation is dose-rate independent [37,38]. The ^137^Cs study γ-H2AX data suggest that peak lymphocyte death occurs following an accumulative ^137^Cs total body committed absorbed dose of ~ 4 Gy, achieved 5 days after isotope administration. Future studies should look to correlate γ-H2AX yields with complete blood count (CBC) measurements and the turnover of specific T-cell subsets following protracted radionuclide exposure.

In a recently published companion study which measured gene expression transcripts in the same peripheral blood mouse samples exposed to ^137^Cs, Paul et al., [26], reported fewer differentially expressed genes compared to the earlier and later time points at the accrued dose of 4.1 Gy (Day 5), suggesting a general transition point at or around this time since the beginning of exposure. Transcriptomic measurements after low-dose ^90^Sr exposure [29] reported that 8082 genes were affected overall, the majority of which were down-regulated with at least 50% overlap between the successive time points, implying that the effect of ^90^Sr was significant throughout the study. Functional analyses were largely related to immune responses, activation of apoptosis of B and T cells, changes in spleen growth, and defects in the response to infection.

Our mechanistic interpretation for the two-peak temporal pattern of γ-H2AX fluorescence assumes two populations of cells; the γ-H2AX signal is initially observed in differentiated mature lymphocytes whereas the second, delayed peak represents DNA damage in newly formed cells. Recent studies have identified that γ-H2AX can also play an active role in transcription [39,40]. Therefore, it is possible that the γ-H2AX kinetic profile observed in this study may not be entirely due to DNA damage response, but also due to changes in transcription and other cellular processes. Although our companion low-dose ^90^Sr blood transcriptomics study showed no significant changes in the mouse H2AFX gene at the mRNA level over the same 30-day time course (Shanaz Ghandhi personal communication), it is possible that the broad changes in the gene levels may contribute to the γ-H2AX response. The finding that γ-H2AX could be good predictive biomarker for damage to the hematopoietic system after radionuclide ingestion also has translational importance. In recent years, the γ-H2AX biomarker has become a powerful tool to monitor DNA DSBs in translational cancer research studies [41–43]. The evaluation of γ-H2AX levels may not only allow for the monitoring of the efficiency of anticancer treatment but also predict tumor cell sensitivity to DNA damaging anticancer agents and toxicity of anticancer treatment toward normal cells [44].

## Conclusions

A key finding in this work was that the γ-H2AX biomarker could be used to measure *in vivo* DNA damage in peripheral blood lymphocytes following the administration of a single radionuclide exposure. The presence of a strong signal several weeks after the start of an internal emitter exposure, is in clear contrast to γ-H2AX signal after acute exposure which is typically undetectable after ~24–48 hours depending on dose [22,45–47]. The γ-H2AX signals for both radionuclides at late times, where the daily dose rate is very low, are larger than would be predicted from the acute-exposure data. Given the different biokinetics of ^137^Cs and ^90^Sr, a mathematical biophysical model was used to analyze to model the data, describing the interplay between increasing cumulative dose, decreasing dose rate during exposure, DSB repair, death of differentiated lymphocyte cells, and production of new lymphocytes from damaged progenitor cells. Ongoing, targeted studies are needed to refine and validate this model, which can in principle be applied to the human experience, given knowledge of the radioisotope biokinetics [15,48].
